# Supplementary material for: Anti-Inflammatory Effects of Spirodela polyrhiza (L.) SCHLEID. Extract on Contact Dermatitis in Mice—Its Active Compounds and Molecular Targets
Source: Int J Mol Sci. 2023 Aug 26;24(17):13271. doi: 10.3390/ijms241713271 (PMC10488168; doi:10.3390/ijms241713271)
Supplement: Supplementary file 1 [file ijms-24-13271-s001.zip › Supplementary S1. Total compounds of S. polyrhiza.pdf]

Supplementary Data S1

Table S1. Total compounds of *S. polyrhiza*

No.	Molecule Name	PubChem CID	Source		
			TCMBank	ETCM	TCMSP
1	(+)-armepavine	680292	○		
2	(10r)-hydroxyhexadeca-7z,11e,13z-trienoicacid	-	○	○	
3	(E,7S,11R)-3,7,11,15-tetramethylhexadec-2-en-1-ol	6474938	○		○
4	(R)-N-methylcoclaurine	440595	○		
5	(R,7E,11Z,13Z)-10-hydroxyhexadeca-7,11,13-trienoic acid	-	○	○	○
6	1,3-phytadiene	-	○		○
7	10-nonacosanol	342803	○		
8	11-hexadecenoic acid	5312414	○		
9	1-o-beta-d-glucopytanosylamplexine	-	○		
10	2-hexadecenoic acid	5282743	○		○
11	2-hydroxychrysophanol	442759	○		
12	2Z-hexadecenoic acid	5312417		○	
13	3-hexadecenoic acid	5312418	○		○
14	4(R)-4-hydroxyisophytol	-	○	○	○
15	5-o-caffeoylquinic acid	5280633			○
16	6-methylcodeine	5492619	○		
17	anonaine	160597	○		
18	apigenin	5280443	○		○
19	apigetrin	5280704	○		○
20	apiose	12306753	○		
21	asparagine	6267			○
22	beta-carotene	5280489	○		○
23	beta-sitosterol	222284		○	○

24	butylated hydroxytoluene	31404	○		○
25	chlorogenic Acid	1794427		○	
26	D-ascorbic acid	54690394	○		○
27	delta-hydrooxylysine	22712964	○	○	
28	glycerol palmitate	14900			○
29	higenamine	114840	○		
30	hypolaetin-8-glucoside	5318255	○	○	
31	isoliensinine	5274591	○		
32	isoquercitrin	51402807	○		
33	isoscoparin	442611	○	○	
34	isoscutellarein	5281665	○		
35	isovitexin	162350	○		○
36	liensinine	160644	○		
37	linoelaidic acid	5282457		○	○
38	linoleate	5460332		○	
39	linolenic acid	5280934		○	○
40	lirinidine	31069	○		
41	lutein	5281243	○		○
42	lutein 5,6-epoxide	5281244	○		○
43	luteolin	5280445	○		○
44	luteolin-7-O-galactoside	5488493		○	○
45	luteolin-7-o-glucoside	5280637	○	○	○
46	lycopersene	5365816	○	○	○
47	methylazoxymethanol	6433205	○		
48	micheline B	10144	○		
49	neferine	134693217	○		
50	nelumboside	11968794	○		
51	nematocyphol acetate	78385349	○		
52	neoxanthin	139033583	○		○

53	n-methylasimilobine	5319512	○		
54	norarmepavine	6999736	○		
55	nornuciferine	624491	○		
56	nuciferine	10146	○		
57	nudicauline	101631701	○		
58	orientin	5281675	○		○
59	palmitic acid	985			○
60	phenol	996	○	○	○
61	propanethial s-oxide	441491	○		
62	quercetin-3-O-galactoside	90657624	○		
63	roemerine	119204	○		
64	rolliniastatin 1	101993683	○		
65	roughanic acid	5312428	○	○	○
66	rutin	5280805	○	○	○
67	safflorye llow	78076204	○		
68	sitogluside	5742590	○		○
69	stigmasterol	5280794	○	○	○
70	tannins	250395	○		
71	trans-1,3-phytodiene	53423857	○		
72	vitexin	5280441	○		○
73	XAT	51346155	○		○
	Total		62	19	34

The compound list was obtained from TCM databases (TCMBank (https://tcmbank.cn/), ETCM v2.0 (http://www.tcmip.cn/ETCM2/front/#/) and TCMSP (https://tcmsp-e.com/tcmsp.php)).
