# Supplementary material for: Anti-Inflammatory Effects of Spirodela polyrhiza (L.) SCHLEID. Extract on Contact Dermatitis in Mice—Its Active Compounds and Molecular Targets
Source: Int J Mol Sci. 2023 Aug 26;24(17):13271. doi: 10.3390/ijms241713271 (PMC10488168; doi:10.3390/ijms241713271)
Supplement: Supplementary file 1 [file ijms-24-13271-s001.zip › Supplementary S3. 29 CD related targets of S. polyrhiza.pdf]

Supplementary data S3

Table S3. 29 CD-related targets of *S. polyrhiza*

No.	Gene Name	Gene_ID	UniProt ID	Full_Name	Protein_Class	EI_{gda}
1	AHR	196	P35869	aryl hydrocarbon receptor	Transcription factor	1
2	BCL2	596	P10415	BCL2 apoptosis regulator	Signaling	1
3	CASP8	841	Q14790	caspase 8	Enzyme	1
4	CCL2	6347	P13500	C-C motif chemokine ligand 2	Signaling	1
5	CCN2	1490	P29279	cellular communication network factor 2	Signaling	1
6	CYP1A1	1543	P04798	cytochrome P450 family 1 subfamily A member 1	Enzyme	1
7	DDIT3	1649	P35638	DNA damage inducible transcript 3		1
8	EPHB2	2048	P29323	EPH receptor B2	Kinase	1
9	GCLC	2729	P48506	glutamate-cysteine ligase catalytic subunit	Enzyme	1
10	GSR	2936	P00390	glutathione-disulfide reductase	Enzyme	1
11	GSTP1	2950	P09211	glutathione S-transferase pi 1		1
12	HMOX1	3162	P09601	heme oxygenase 1		1
13	IFNG	3458	P01579	interferon gamma		1
14	IL1A	3552	P01583	interleukin 1 alpha		1
15	IL1B	3553	P01584	interleukin 1 beta		1
16	IL4	3565	P05112	interleukin 4		1
17	IL6	3569	P05231	interleukin 6		1
18	MAPK1	5594	P28482	mitogen-activated protein kinase 1	Kinase	1
19	MMP10	4319	P09238	matrix metallopeptidase 10	Enzyme	1
20	OPRM1	4988	P35372	opioid receptor mu 1	G-protein coupled receptor	1
21	S100A8	6279	P05109	S100 calcium binding protein A8	Calcium-binding protein	1

22	SOD1	6647	P00441	superoxide dismutase 1	Enzyme	1
23	SOD3	6649	P08294	superoxide dismutase 3	Enzyme	1
24	TLR4	7099	O00206	toll like receptor 4		1
25	TNF	7124	P01375	tumor necrosis factor	Signaling	0.8
26	TPSAB1	7177	P20231;Q15661	tryptase alpha/beta 1	Enzyme	1
27	UGT1A1	54658	P22309	UDP glucuronosyltransferase family 1 member A1		1
28	UGT1A6	54578	P19224	UDP glucuronosyltransferase family 1 member A6		1
29	VEGFA	7422	P15692	vascular endothelial growth factor A	Signaling	1

EL, Evidence Index.
