# Supplementary material for: Anti-Inflammatory Effects of Spirodela polyrhiza (L.) SCHLEID. Extract on Contact Dermatitis in Mice—Its Active Compounds and Molecular Targets
Source: Int J Mol Sci. 2023 Aug 26;24(17):13271. doi: 10.3390/ijms241713271 (PMC10488168; doi:10.3390/ijms241713271)
Supplement: Supplementary file 1 [file ijms-24-13271-s001.zip › Supplementary S4. Key protein prediction results.pdf]

Supplementary data S4

Table S4. Hub gene prediction results among 29 CD-related target genes

Ranking	MCC		EPC		MNC		Degree	
1	IL6	5810	TNF	18.292	IL6	11	IL6	11
2	TNF	5808	IL1B	18.284	TNF	10	TNF	10
3	IL1B	5808	IL4	18.272	IL1B	10	IL1B	10
4	IL4	5784	IL6	18.271	IL4	9	IL4	9
5	CCL2	5760	TLR4	18.225	CCL2	8	CCL2	8
6	IL1A	5760	CCL2	18.22	IL1A	8	IL1A	8
7	TLR4	5065	IL1A	18.204	TLR4	8	TLR4	8
8	IFNG	5040	IFNG	18.138	IFNG	7	IFNG	7
9	VEGFA	721	VEGFA	18.105	CASP8	6	CASP8	6
10	HMOX1	36	HMOX1	17.914	VEGFA	6	VEGFA	6
11	CASP8	28	CASP8	17.762	GSR	5	GSR	5
12	GSR	14	SOD1	16.898	HMOX1	4	HMOX1	4
13	SOD1	13	GSR	16.374	GCLC	4	GCLC	4
14	GCLC	8	GCLC	15.96	SOD1	4	SOD1	4
15	SOD3	6	SOD3	15.388	MAPK1	3	MAPK1	3
16	MAPK1	4	BCL2	15.384	SOD3	3	SOD3	3
17	CYP1A1	4	MAPK1	15.299	BCL2	2	BCL2	2
18	BCL2	4	GSTP1	12.889	GSTP1	2	GSTP1	2
19	GSTP1	3	CTGF	9.801	AHR	1	AHR	1
20	AHR	1	S100A8	9.627	UGT1A6	1	UGT1A6	1
21	UGT1A6	1	CYP1A1	8.331	CTGF	1	CTGF	1
22	CTGF	1	DDIT3	8.298	S100A8	1	S100A8	1
23	S100A8	1	UGT1A6	4.895	UGT1A1	1	UGT1A1	1
24	UGT1A1	1	UGT1A1	4.704	CYP1A1	1	CYP1A1	1

25	DDIT3	1	AHR	4.553	DDIT3	1	DDIT3	1
26	MMP10	0	MMP10	1	MMP10	0	MMP10	0
27	TPSAB1	0	TPSAB1	1	TPSAB1	0	TPSAB1	0
28	EPHB2	0	EPHB2	1	EPHB2	0	EPHB2	0
29	OPRM1	0	OPRM1	1	OPRM1	0	OPRM1	0

The STRING database and the Cytoscape plugin CytoHubba was used for the prediction of hub genes from the PPI analysis. MCC, maximal clique centrality; EPC, edge percolated component; MNC, maximum neighborhood component; Degree, node connect degree.
