# Supplementary material for: Anti-Inflammatory Effects of Spirodela polyrhiza (L.) SCHLEID. Extract on Contact Dermatitis in Mice—Its Active Compounds and Molecular Targets
Source: Int J Mol Sci. 2023 Aug 26;24(17):13271. doi: 10.3390/ijms241713271 (PMC10488168; doi:10.3390/ijms241713271)
Supplement: Supplementary file 1 [file ijms-24-13271-s001.zip › Supplementary S5. Infiltrated immune cells.pdf]

Supplementary data S5

Evaluations of immune cell infiltration

Cells were enumerated using a cell counting grid (2.1 × 1.6 mm, 100x magnification) to evaluate immune cell infiltration in four randomly selected, non-overlapping regions per slide. Immune cells were defined as macrophages, polymorphonuclear leukocytes, lymphocytes, eosinophils, plasma cells, and giant cells. The number of infiltrated immune cells was represented as number per square millimeter.

EESP prevented DNFB-induced immune cell infiltration.

Repeated application of DNFB resulted in immune cell infiltration around dermis and blood vessels. Most of the infiltrated immune cells were neutrophils or lymphocytes. EESP administration at 150 and 500 μg/day considerably reduced these infiltrating immune cell numbers.

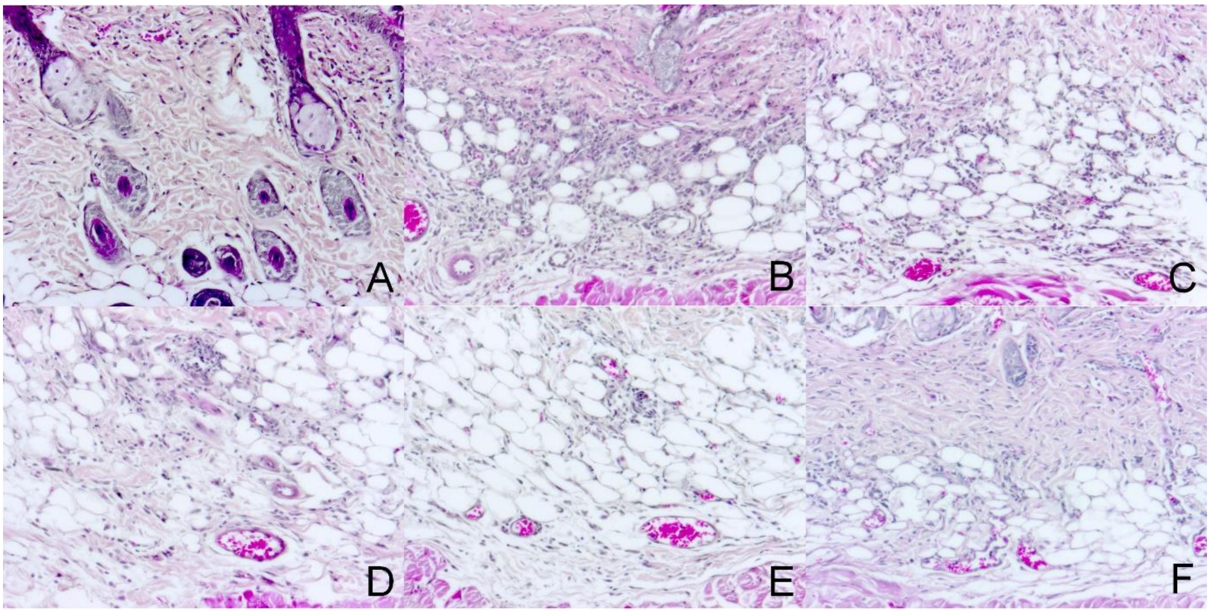

Figure S1. Effects of EESP on numbers of infiltrating immune cells A, Treatment naïve (NOR); B, CD control (CTL); C, 50 μg/day EESP; D, 150 μg/day EESP; E, 500 μg/day EEFR; and F, 150 μg/day DEX (Original magnification x100).
