# Supplementary material for: Anti-Inflammatory Effects of Spirodela polyrhiza (L.) SCHLEID. Extract on Contact Dermatitis in Mice—Its Active Compounds and Molecular Targets
Source: Int J Mol Sci. 2023 Aug 26;24(17):13271. doi: 10.3390/ijms241713271 (PMC10488168; doi:10.3390/ijms241713271)
Supplement: Supplementary file 1 [file ijms-24-13271-s001.zip › Supplementary S6. RNA-seq Automatic GEO signatures.pdf]

Supplementary data S6

The identification of enriched transcriptomic signatures

For the identification of enriched transcriptomic signatures, analysis was conducted by using Enrichr website (https://maayanlab.cloud/Enrichr/).

Target genes had a significant association with the gene set linked to atopic dermatitis

The enrichment analysis of RNAseq automatic Gene Expression Omnibus (GEO) signatures revealed that active compounds of *S. polyrhiza* (730 target genes) were associated with mouse down gene set including Atopic Dermatitis Nestin-Cre IKK2 (GSE109936).

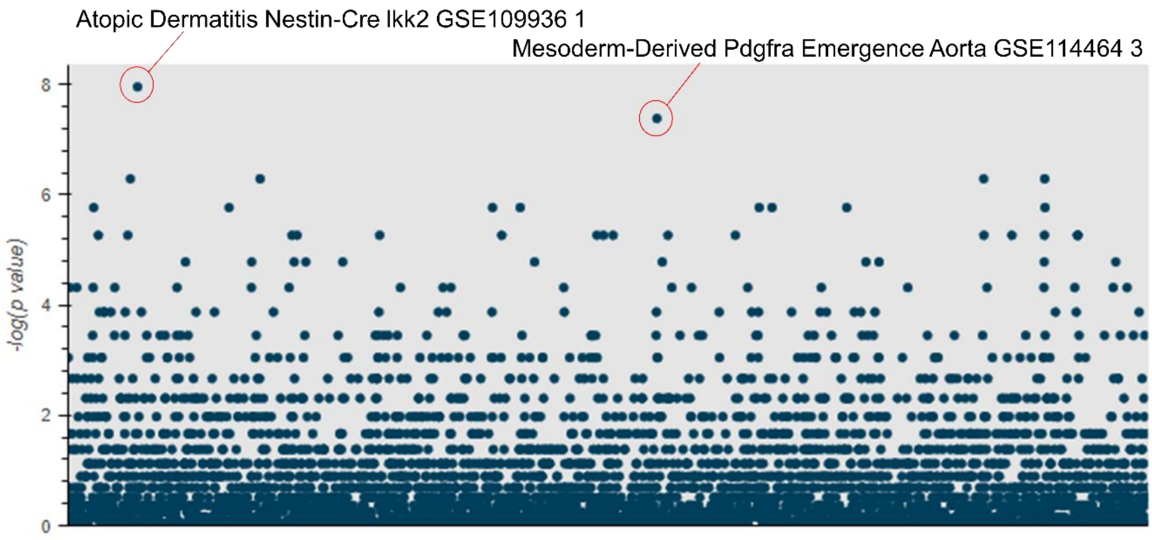

Figure S2. Manhattan plot of terms from the RNA-seq_Automatic_GEO_Signatures_Mouse_Down gene set. Each point represents a single term along the x-axis. The y-values represent the -log10 (p-value) corresponding to the enrichment of the input gene set for the term gene set. Hovering over the point will display the gene set term and the enrichment analysis *p*-value.
