# Supplementary material for: Anti-Inflammatory Effects of Spirodela polyrhiza (L.) SCHLEID. Extract on Contact Dermatitis in Mice—Its Active Compounds and Molecular Targets
Source: Int J Mol Sci. 2023 Aug 26;24(17):13271. doi: 10.3390/ijms241713271 (PMC10488168; doi:10.3390/ijms241713271)
Supplement: Supplementary file 1 [file ijms-24-13271-s001.zip › Supplementary S7. Changes in BW and SB ratio.pdf]

Supplementary data S7

Measurement of body and spleen weights

Body weights of each mouse were measured on day 1 and day 15 using electronic scale (CAS, Gyeonggi, Republic of Korea) respectively. Changes in body weights were expressed as percentages of weight on day 1. Spleen weights were measured on day 15 using microbalance (Sartorius, Gyeonggi, Republic of Korea). The effects of EESP on spleen weights are presented as the spleen body weight ratio.

EESP did not affect changes in body weights and spleen / body weight ratio.

Topical application of EESP did not affect changes in body weights unlike dexamethasone (DEX). DEX significantly suppressed weight gain compared to those of the CTL and the NOR group (Figure S3A). The effect of EESP on spleen enlargement was estimated by determining spleen/body weight ratio. The inflammatory response induced by DNFB stimulation caused spleen enlargement. The spleen/body weight ratios in the EESP treated groups were similar to that of the CTL group. DEX treatment significantly lowered spleen/body weight ratio compared to that of the CTL as well as the NOR group (Figure S3B).

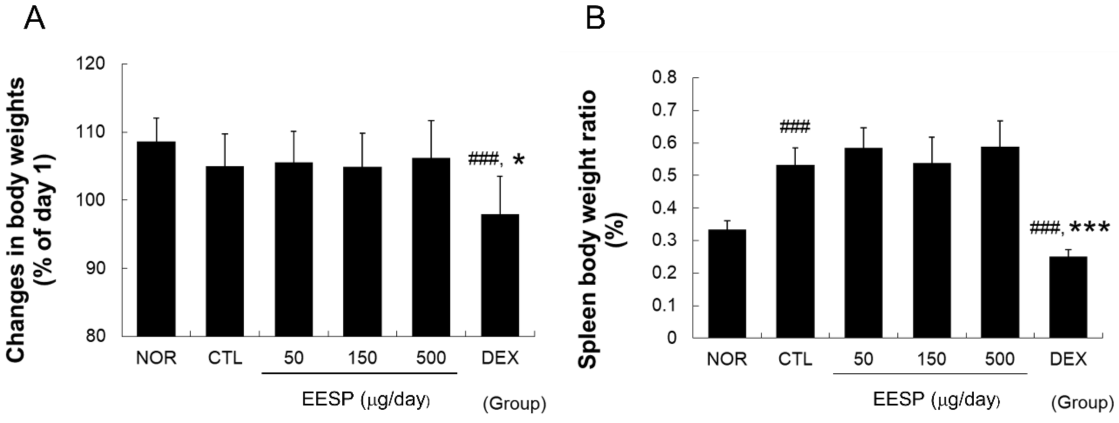

Figure S3. Effects of EESP on changes in body weights and spleen/body weight ratio in CD mice. Body and spleen weight were measured on day 15 and the spleen/body weight ratio was calculated. (A), changes in body weights; (B), spleen/body weight ratio. $^{###}p < 0.001$ vs. NOR; $^{*}p < 0.05$ and $^{***}p < 0.001$ vs. CTL.
