# Supplementary material for: Anti-Inflammatory Effects of Spirodela polyrhiza (L.) SCHLEID. Extract on Contact Dermatitis in Mice—Its Active Compounds and Molecular Targets
Source: Int J Mol Sci. 2023 Aug 26;24(17):13271. doi: 10.3390/ijms241713271 (PMC10488168; doi:10.3390/ijms241713271)
Supplement: Supplementary file 1 [file ijms-24-13271-s001.zip › Supplementary S9. Experimental schedule.pdf]

Supplementary data S9

CD induction and experimental schedule

Mice were sensitized with 30 μL of DNFB (0.2%, *v/v*) in acetone: olive oil (AOO, 4:1) onto both ears for three consecutive days. After four days of sensitization, the dorsum of all mice were shaved. On day 8, each mouse was challenged with 60 μL of DNFB (0.1% *v/v*) in AOO onto the shaved dorsum every two days (four times applications in total).

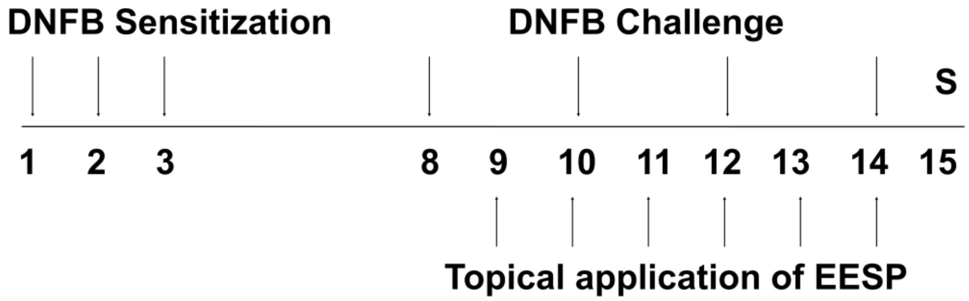

Figure S5. Experimental schedule. DNFB was applied onto the surface of each ear for sensitization on days 1, 2 and 3. Challenging was conducted using 0.1% of DNFB on days 8, 10, 12 and 14. EESP and dexamethasone were topically applied for 6 consecutive days. All animals were sacrificed on day 15. EESP, 70% ethanol extract of *S. polyrhiza* whole plant; DNFB, 1-fluoro-2,4-dinitrobenzene; S, sacrifice.
