# Supplementary figures and images for: Anti-Inflammatory Effects of Spirodela polyrhiza (L.) SCHLEID. Extract on Contact Dermatitis in Mice—Its Active Compounds and Molecular Targets
Source: Int J Mol Sci. 2023 Aug 26;24(17):13271. doi: 10.3390/ijms241713271 (PMC10488168; doi:10.3390/ijms241713271)

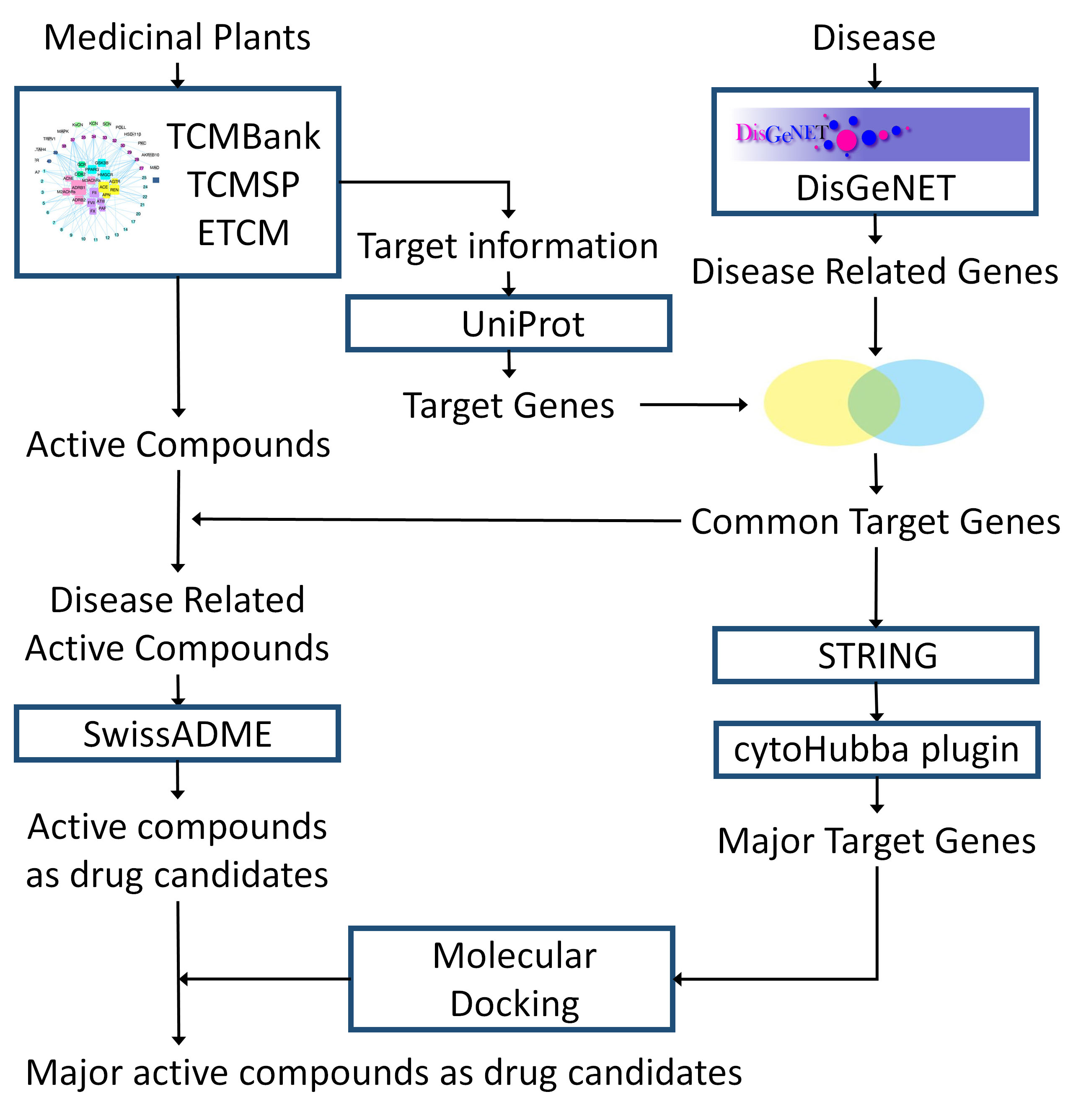

Supplement: Supplementary file 1 [file ijms-24-13271-s001.zip › Supplementary S8. Flow of network based analysis (Figure S4).jpg]
